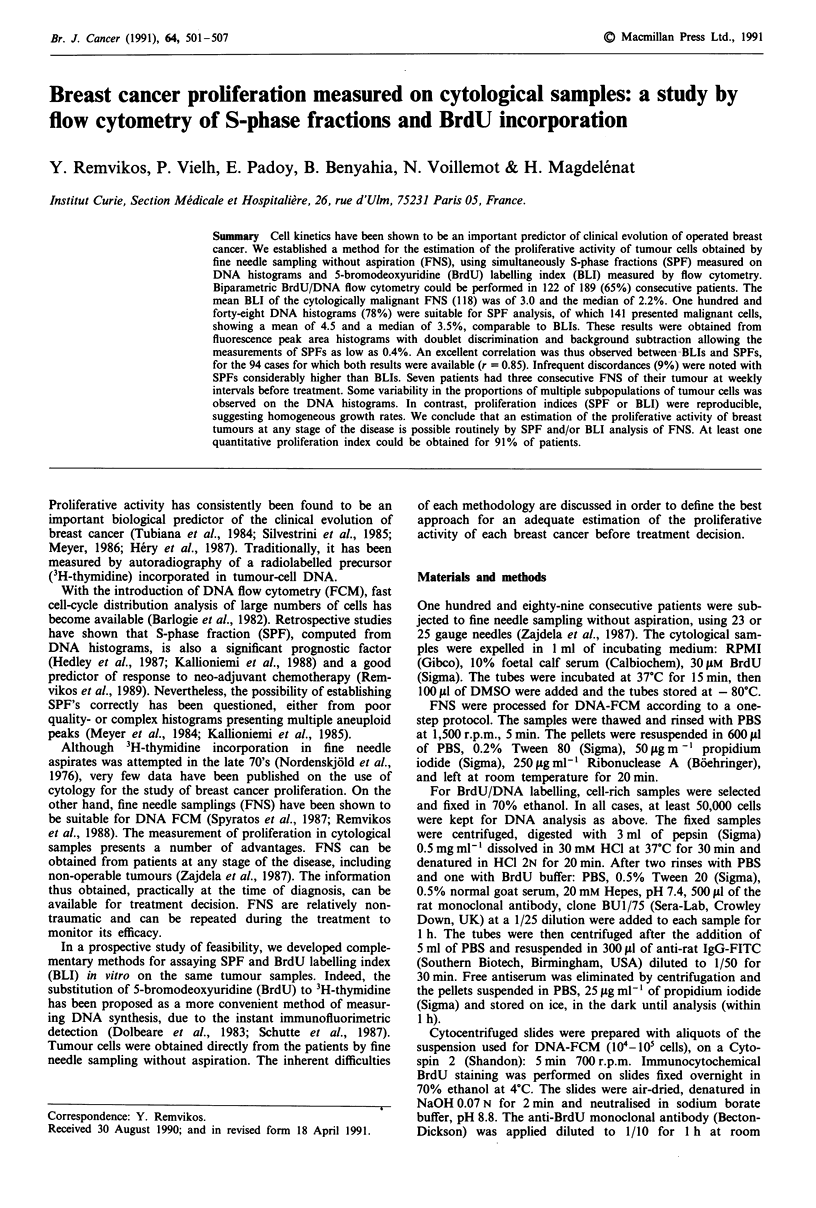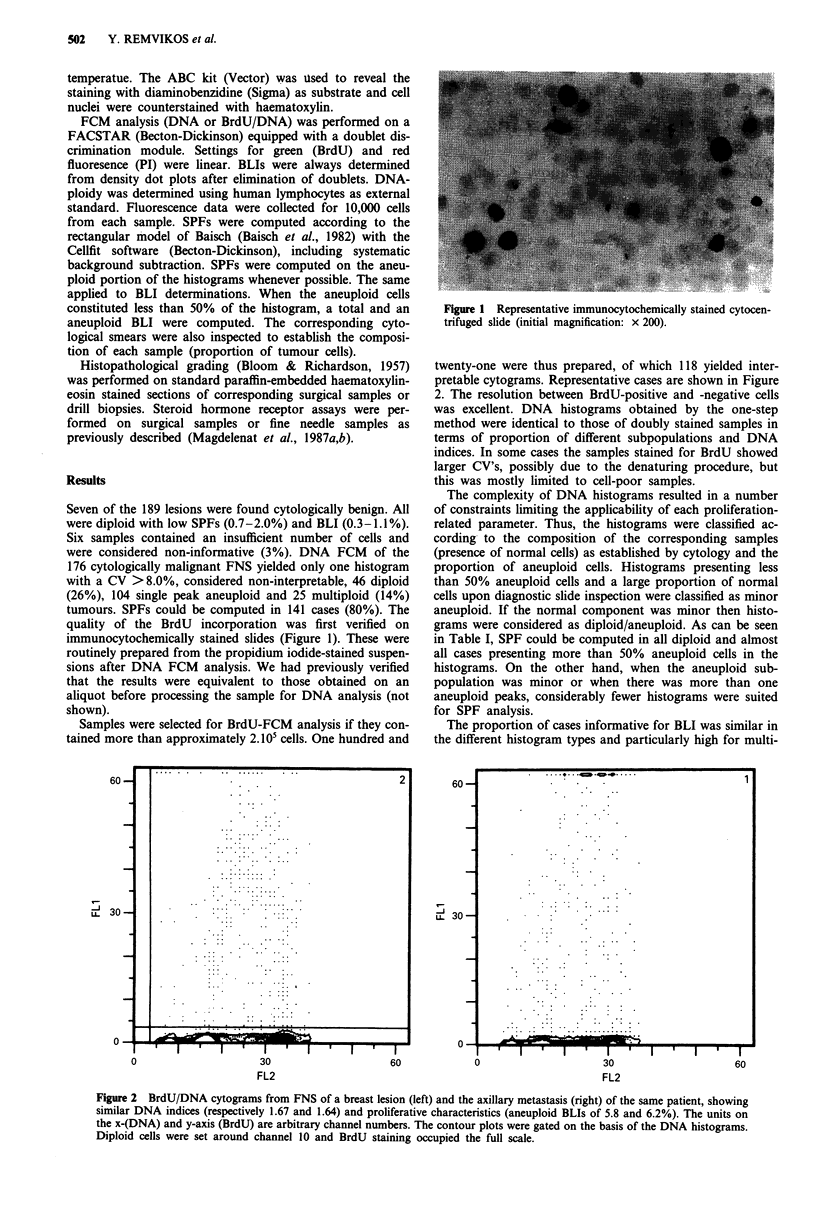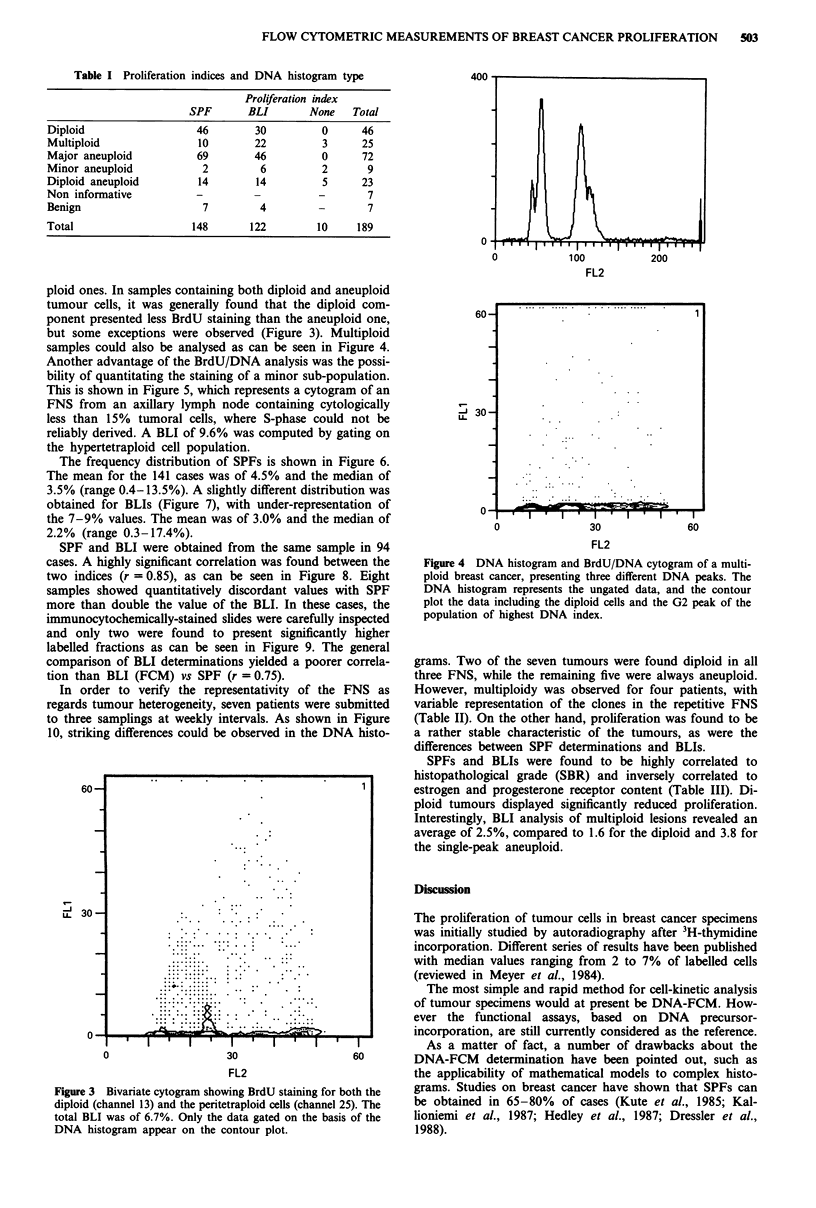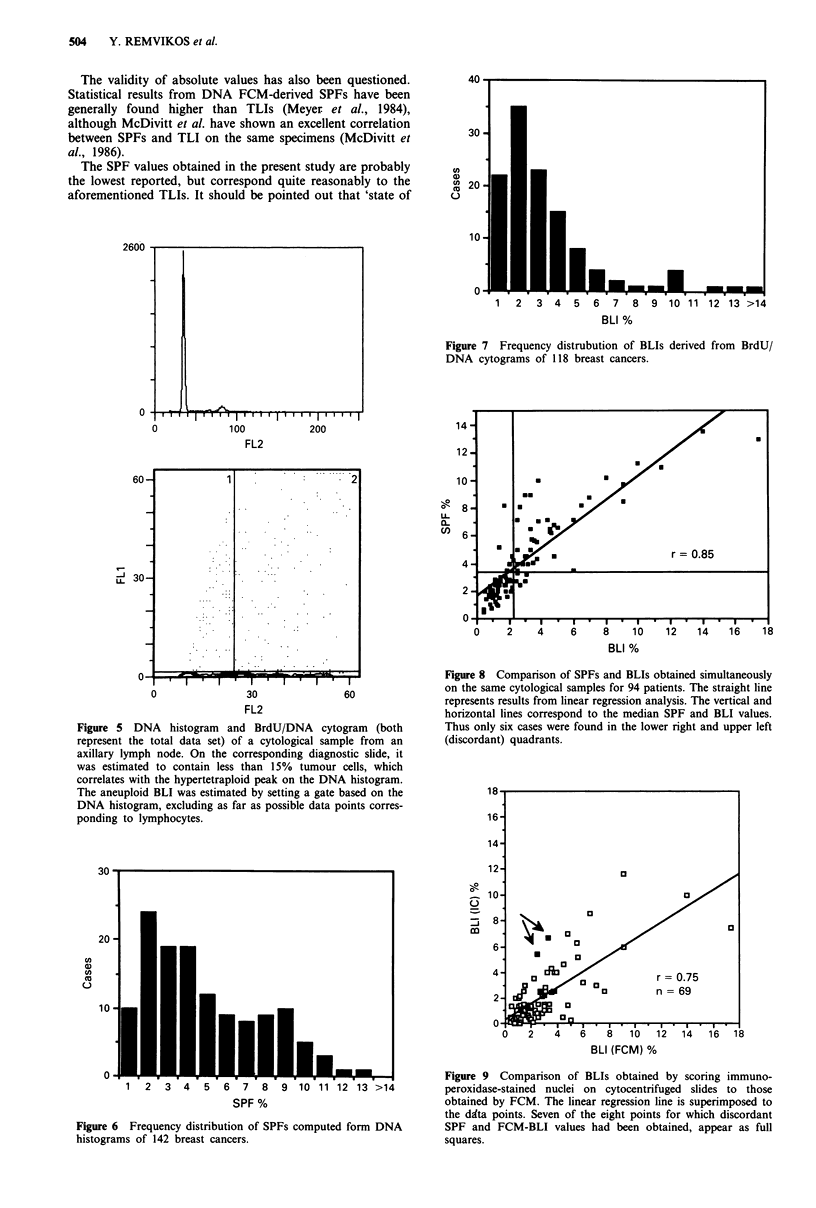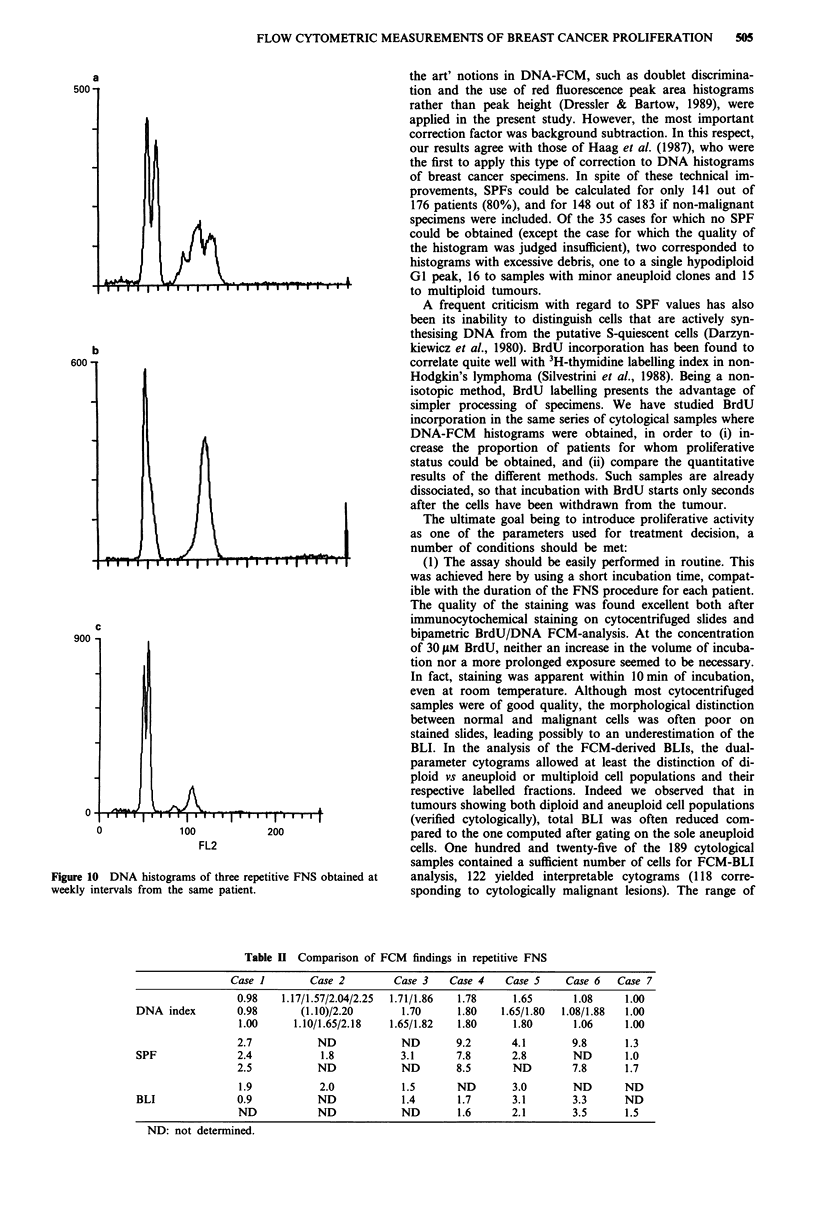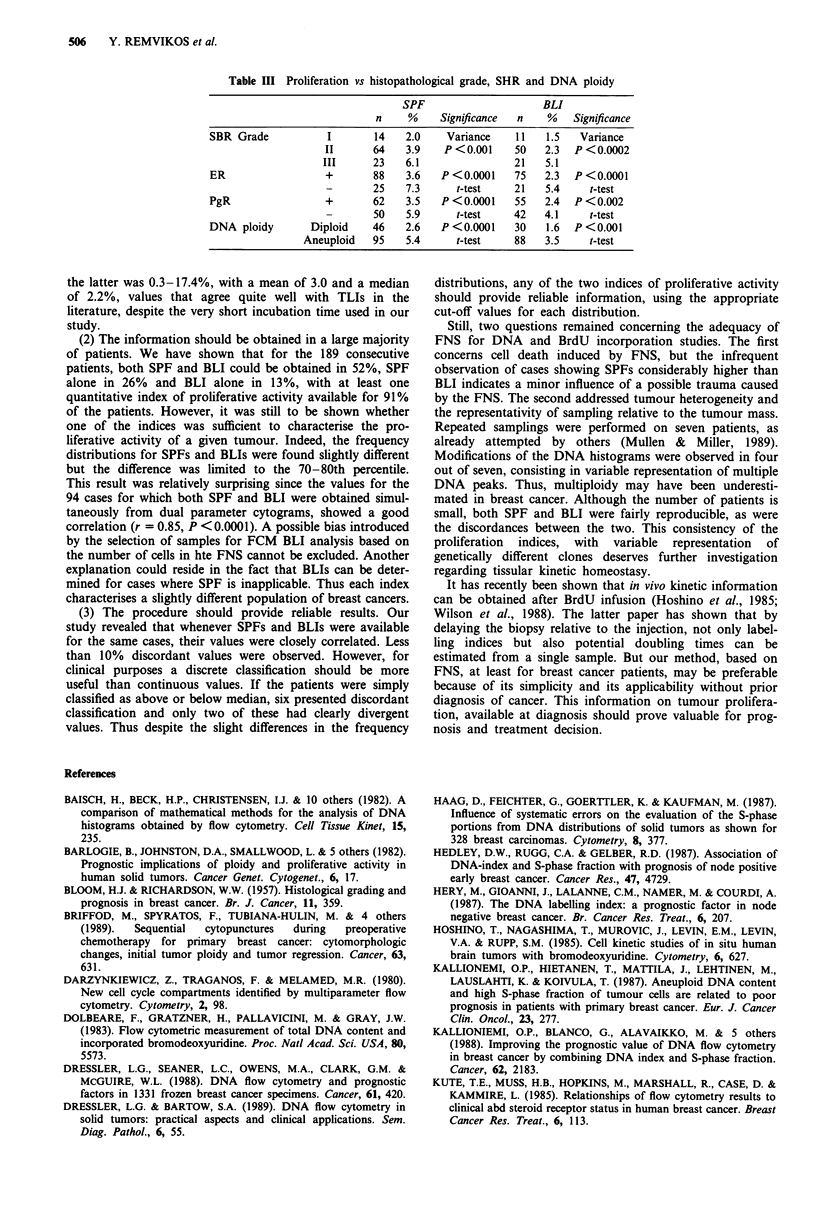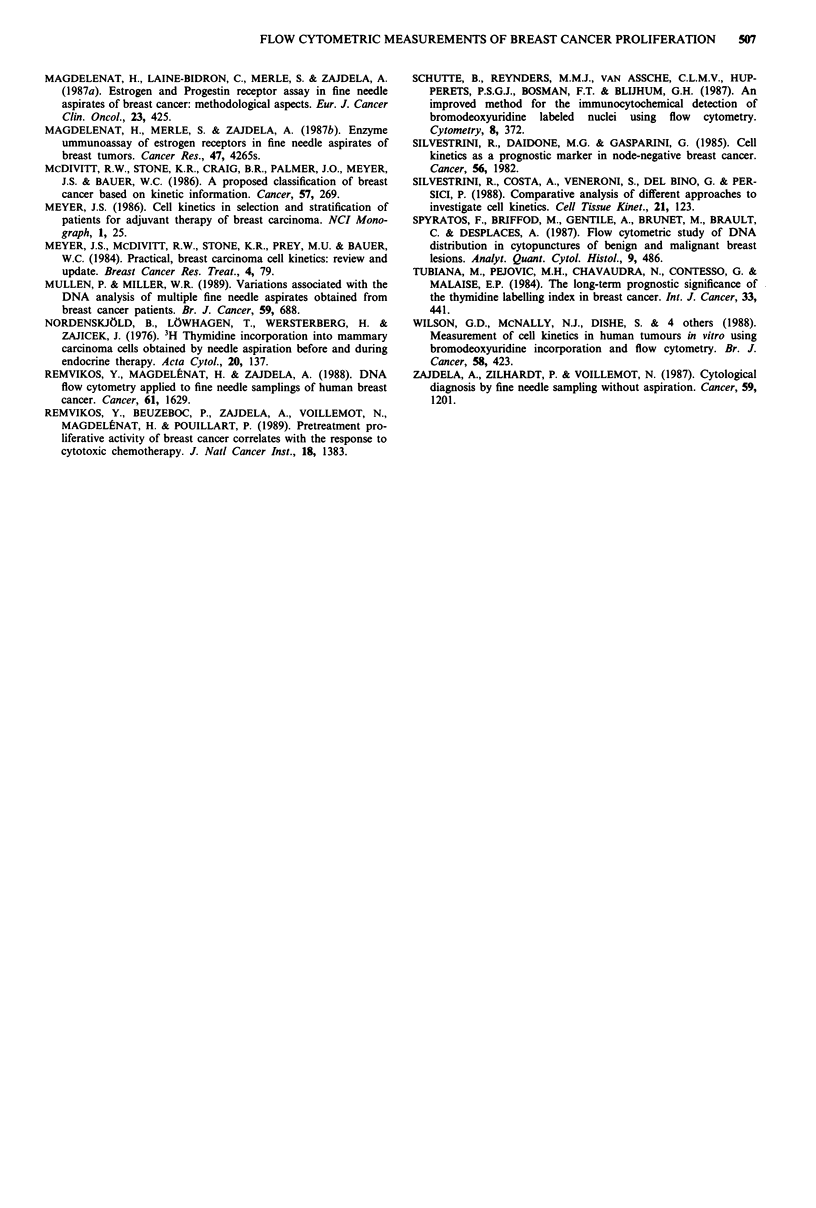# Breast cancer proliferation measured on cytological samples: a study by flow cytometry of S-phase fractions and BrdU incorporation.

**DOI:** 10.1038/bjc.1991.338

**Published:** 1991-09

**Authors:** Y. Remvikos, P. Vielh, E. Padoy, B. Benyahia, N. Voillemot, H. Magdelénat

**Affiliations:** Institut Curie, Section Médicale et Hospitalière, Paris, France.

## Abstract

**Images:**


Br. J. Cancer (1991), 64, 501-507 © Macmillan Press Ltd., 1991

### Breast cancer proliferation measured on cytological samples: a study by flow cytometry of S-phase fractions and BrdU incorporation

Y. Remvikos, P. Vielh, E. Padoy, B. Benyahia, N. Voillemot & H. Magdelénat

*Institut Curie, Section Médicale et Hospitalière, 26, rue d'Ulm, 75231 Paris 05, France.*

**Summary** Cell kinetics have been shown to be an important predictor of clinical evolution of operated breast cancer. We established a method for the estimation of the proliferative activity of tumour cells obtained by fine needle sampling without aspiration (FNS), using simultaneously S-phase fractions (SPF) measured on DNA histograms and 5-bromodeoxyuridine (BrdU) labelling index (BLI) measured by flow cytometry. Biparametric BrdU/DNA flow cytometry could be performed in 122 of 189 (65%) consecutive patients. The mean BLI of the cytologically malignant FNS (118) was of 3.0 and the median of 2.2%. One hundred and forty-eight DNA histograms (78%) were suitable for SPF analysis, of which 141 presented malignant cells, showing a mean of 4.5 and a median of 3.5%, comparable to BLIs. These results were obtained from fluorescence peak area histograms with doublet discrimination and background subtraction allowing the measurements of SPFs as low as 0.4%. An excellent correlation was thus observed between BLIs and SPFs, for the 94 cases for which both results were available ($r = 0.85$). Infrequent discordances (9%) were noted with SPFs considerably higher than BLIs. Seven patients had three consecutive FNS of their tumour at weekly intervals before treatment. Some variability in the proportions of multiple subpopulations of tumour cells was observed on the DNA histograms. In contrast, proliferation indices (SPF or BLI) were reproducible, suggesting homogeneous growth rates. We conclude that an estimation of the proliferative activity of breast tumours at any stage of the disease is possible routinely by SPF and/or BLI analysis of FNS. At least one quantitative proliferation index could be obtained for 91% of patients.

Proliferative activity has consistently been found to be an important biological predictor of the clinical evolution of breast cancer (Tubiana *et al.*, 1984; Silvestrini *et al.*, 1985; Meyer, 1986; Héry *et al.*, 1987). Traditionally, it has been measured by autoradiography of a radiolabelled precursor ($^3$H-thymidine) incorporated in tumour-cell DNA.

With the introduction of DNA flow cytometry (FCM), fast cell-cycle distribution analysis of large numbers of cells has become available (Barlogie *et al.*, 1982). Retrospective studies have shown that S-phase fraction (SPF), computed from DNA histograms, is also a significant prognostic factor (Hedley *et al.*, 1987; Kallioniemi *et al.*, 1988) and a good predictor of response to neo-adjuvant chemotherapy (Remvikos *et al.*, 1989). Nevertheless, the possibility of establishing SPF's correctly has been questioned, either from poor quality- or complex histograms presenting multiple aneuploid peaks (Meyer *et al.*, 1984; Kallioniemi *et al.*, 1985).

Although $^3$H-thymidine incorporation in fine needle aspirates was attempted in the late 70's (Nordenskjöld *et al.*, 1976), very few data have been published on the use of cytology for the study of breast cancer proliferation. On the other hand, fine needle samplings (FNS) have been shown to be suitable for DNA FCM (Spyratos *et al.*, 1987; Remvikos *et al.*, 1988). The measurement of proliferation in cytological samples presents a number of advantages. FNS can be obtained from patients at any stage of the disease, including non-operable tumours (Zajdela *et al.*, 1987). The information thus obtained, practically at the time of diagnosis, can be available for treatment decision. FNS are relatively non-traumatic and can be repeated during the treatment to monitor its efficacy.

In a prospective study of feasibility, we developed complementary methods for assaying SPF and BrdU labelling index (BLI) *in vitro* on the same tumour samples. Indeed, the substitution of 5-bromodeoxyuridine (BrdU) to $^3$H-thymidine has been proposed as a more convenient method of measuring DNA synthesis, due to the instant immunofluorimetric detection (Dolbeare *et al.*, 1983; Schutte *et al.*, 1987). Tumour cells were obtained directly from the patients by fine needle sampling without aspiration. The inherent difficulties of each methodology are discussed in order to define the best approach for an adequate estimation of the proliferative activity of each breast cancer before treatment decision.

### Materials and methods

One hundred and eighty-nine consecutive patients were subjected to fine needle sampling without aspiration, using 23 or 25 gauge needles (Zajdela *et al.*, 1987). The cytological samples were expelled in 1 ml of incubating medium: RPMI (Gibco), 10% foetal calf serum (Calbiochem), 30 µM BrdU (Sigma). The tubes were incubated at 37°C for 15 min, then 100 µl of DMSO were added and the tubes stored at − 80°C.

FNS were processed for DNA-FCM according to a one-step protocol. The samples were thawed and rinsed with PBS at 1,500 r.p.m., 5 min. The pellets were resuspended in 600 µl of PBS, 0.2% Tween 80 (Sigma), 50 µg m$^{-1}$ propidium iodide (Sigma), 250 µg ml$^{-1}$ Ribonuclease A (Böehringer), and left at room temperature for 20 min.

For BrdU/DNA labelling, cell-rich samples were selected and fixed in 70% ethanol. In all cases, at least 50,000 cells were kept for DNA analysis as above. The fixed samples were centrifuged, digested with 3 ml of pepsin (Sigma) 0.5 mg ml$^{-1}$ dissolved in 30 mM HCl at 37°C for 30 min and denatured in HCl 2N for 20 min. After two rinses with PBS and one with BrdU buffer: PBS, 0.5% Tween 20 (Sigma), 0.5% normal goat serum, 20 mM Hepes, pH 7.4, 500 µl of the rat monoclonal antibody, clone BU1/75 (Sera-Lab, Crowley Down, UK) at a 1/25 dilution were added to each sample for 1 h. The tubes were then centrifuged after the addition of 5 ml of PBS and resuspended in 300 µl of anti-rat IgG-FITC (Southern Biotech, Birmingham, USA) diluted to 1/50 for 30 min. Free antiserum was eliminated by centrifugation and the pellets suspended in PBS, 25 µg ml$^{-1}$ of propidium iodide (Sigma) and stored on ice, in the dark until analysis (within 1 h).

Cytocentrifuged slides were prepared with aliquots of the suspension used for DNA-FCM ($10^4$–$10^5$ cells), on a Cytospin 2 (Shandon): 5 min 700 r.p.m. Immunocytochemical BrdU staining was performed on slides fixed overnight in 70% ethanol at 4°C. The slides were air-dried, denatured in NaOH 0.07 N for 2 min and neutralised in sodium borate buffer, pH 8.8. The anti-BrdU monoclonal antibody (Becton-Dickson) was applied diluted to 1/10 for 1 h at room

Correspondence: Y. Remvikos.
Received 30 August 1990; and in revised form 18 April 1991.

temperatue. The ABC kit (Vector) was used to reveal the staining with diaminobenzidine (Sigma) as substrate and cell nuclei were counterstained with haematoxylin.

FCM analysis (DNA or BrdU/DNA) was performed on a FACSTAR (Becton-Dickinson) equipped with a doublet discrimination module. Settings for green (BrdU) and red fluoresence (PI) were linear. BLIs were always determined from density dot plots after elimination of doublets. DNA-ploidy was determined using human lymphocytes as external standard. Fluorescence data were collected for 10,000 cells from each sample. SPFs were computed according to the rectangular model of Baisch (Baisch et al., 1982) with the Cellfit software (Becton-Dickinson), including systematic background subtraction. SPFs were computed on the aneuploid portion of the histograms whenever possible. The same applied to BLI determinations. When the aneuploid cells constituted less than 50% of the histogram, a total and an aneuploid BLI were computed. The corresponding cytological smears were also inspected to establish the composition of each sample (proportion of tumour cells).

Histopathological grading (Bloom & Richardson, 1957) was performed on standard paraffin-embedded haematoxylin-eosin stained sections of corresponding surgical samples or drill biopsies. Steroid hormone receptor assays were performed on surgical samples or fine needle samples as previously described (Magdelenat et al., 1987a,b).

### Results

Seven of the 189 lesions were found cytologically benign. All were diploid with low SPFs (0.7–2.0%) and BLI (0.3–1.1%). Six samples contained an insufficient number of cells and were considered non-informative (3%). DNA FCM of the 176 cytologically malignant FNS yielded only one histogram with a CV >8.0%, considered non-interpretable, 46 diploid (26%), 104 single peak aneuploid and 25 multiploid (14%) tumours. SPFs could be computed in 141 cases (80%). The quality of the BrdU incorporation was first verified on immunocytochemically stained slides (Figure 1). These were routinely prepared from the propidium iodide-stained suspensions after DNA FCM analysis. We had previously verified that the results were equivalent to those obtained on an aliquot before processing the sample for DNA analysis (not shown).

Samples were selected for BrdU-FCM analysis if they contained more than approximately $2.10^5$ cells. One hundred and twenty-one were thus prepared, of which 118 yielded interpretable cytograms. Representative cases are shown in Figure 2. The resolution between BrdU-positive and -negative cells was excellent. DNA histograms obtained by the one-step method were identical to those of doubly stained samples in terms of proportion of different subpopulations and DNA indices. In some cases the samples stained for BrdU showed larger CV's, possibly due to the denaturing procedure, but this was mostly limited to cell-poor samples.

**Figure 1** Representative immunocytochemically stained cytocentrifuged slide (initial magnification: × 200).

The complexity of DNA histograms resulted in a number of constraints limiting the applicability of each proliferation-related parameter. Thus, the histograms were classified according to the composition of the corresponding samples (presence of normal cells) as established by cytology and the proportion of aneuploid cells. Histograms presenting less than 50% aneuploid cells and a large proportion of normal cells upon diagnostic slide inspection were classified as minor aneuploid. If the normal component was minor then histograms were considered as diploid/aneuploid. As can be seen in Table I, SPF could be computed in all diploid and almost all cases presenting more than 50% aneuploid cells in the histograms. On the other hand, when the aneuploid subpopulation was minor or when there was more than one aneuploid peaks, considerably fewer histograms were suited for SPF analysis.

The proportion of cases informative for BLI was similar in the different histogram types and particularly high for multi-

**Figure 2** BrdU/DNA cytograms from FNS of a breast lesion (left) and the axillary metastasis (right) of the same patient, showing similar DNA indices (respectively 1.67 and 1.64) and proliferative characteristics (aneuploid BLIs of 5.8 and 6.2%). The units on the x-(DNA) and y-axis (BrdU) are arbitrary channel numbers. The contour plots were gated on the basis of the DNA histograms. Diploid cells were set around channel 10 and BrdU staining occupied the full scale.

**Table I** Proliferation indices and DNA histogram type

| | Proliferation index | | | |
|---|---|---|---|---|
| | *SPF* | *BLI* | *None* | *Total* |
| Diploid | 46 | 30 | 0 | 46 |
| Multiploid | 10 | 22 | 3 | 25 |
| Major aneuploid | 69 | 46 | 0 | 72 |
| Minor aneuploid | 2 | 6 | 2 | 9 |
| Diploid aneuploid | 14 | 14 | 5 | 23 |
| Non informative | – | – | – | 7 |
| Benign | 7 | 4 | – | 7 |
| Total | 148 | 122 | 10 | 189 |

ploid ones. In samples containing both diploid and aneuploid tumour cells, it was generally found that the diploid component presented less BrdU staining than the aneuploid one, but some exceptions were observed (Figure 3). Multiploid samples could also be analysed as can be seen in Figure 4. Another advantage of the BrdU/DNA analysis was the possibility of quantitating the staining of a minor sub-population. This is shown in Figure 5, which represents a cytogram of an FNS from an axillary lymph node containing cytologically less than 15% tumoral cells, where S-phase could not be reliably derived. A BLI of 9.6% was computed by gating on the hypertetraploid cell population.

The frequency distribution of SPFs is shown in Figure 6. The mean for the 141 cases was of 4.5% and the median of 3.5% (range 0.4–13.5%). A slightly different distribution was obtained for BLIs (Figure 7), with under-representation of the 7–9% values. The mean was of 3.0% and the median of 2.2% (range 0.3–17.4%).

SPF and BLI were obtained from the same sample in 94 cases. A highly significant correlation was found between the two indices ($r = 0.85$), as can be seen in Figure 8. Eight samples showed quantitatively discordant values with SPF more than double the value of the BLI. In these cases, the immunocytochemically-stained slides were carefully inspected and only two were found to present significantly higher labelled fractions as can be seen in Figure 9. The general comparison of BLI determinations yielded a poorer correlation than BLI (FCM) *vs* SPF ($r = 0.75$).

In order to verify the representativity of the FNS as regards tumour heterogeneity, seven patients were submitted to three samplings at weekly intervals. As shown in Figure 10, striking differences could be observed in the DNA histo-

**Figure 3** Bivariate cytogram showing BrdU staining for both the diploid (channel 13) and the peritetraploid cells (channel 25). The total BLI was of 6.7%. Only the data gated on the basis of the DNA histogram appear on the contour plot.

**Figure 4** DNA histogram and BrdU/DNA cytogram of a multiploid breast cancer, presenting three different DNA peaks. The DNA histogram represents the ungated data, and the contour plot the data including the diploid cells and the G2 peak of the population of highest DNA index.

grams. Two of the seven tumours were found diploid in all three FNS, while the remaining five were always aneuploid. However, multiploidy was observed for four patients, with variable representation of the clones in the repetitive FNS (Table II). On the other hand, proliferation was found to be a rather stable characteristic of the tumours, as were the differences between SPF determinations and BLIs.

SPFs and BLIs were found to be highly correlated to histopathological grade (SBR) and inversely correlated to estrogen and progesterone receptor content (Table III). Diploid tumours displayed significantly reduced proliferation. Interestingly, BLI analysis of multiploid lesions revealed an average of 2.5%, compared to 1.6 for the diploid and 3.8 for the single-peak aneuploid.

### Discussion

The proliferation of tumour cells in breast cancer specimens was initially studied by autoradiography after $^3$H-thymidine incorporation. Different series of results have been published with median values ranging from 2 to 7% of labelled cells (reviewed in Meyer *et al.*, 1984).

The most simple and rapid method for cell-kinetic analysis of tumour specimens would at present be DNA-FCM. However the functional assays, based on DNA precursor-incorporation, are still currently considered as the reference.

As a matter of fact, a number of drawbacks about the DNA-FCM determination have been pointed out, such as the applicability of mathematical models to complex histograms. Studies on breast cancer have shown that SPFs can be obtained in 65–80% of cases (Kute *et al.*, 1985; Kallioniemi *et al.*, 1987; Hedley *et al.*, 1987; Dressler *et al.*, 1988).

The validity of absolute values has also been questioned. Statistical results from DNA FCM-derived SPFs have been generally found higher than TLIs (Meyer et al., 1984), although McDivitt et al. have shown an excellent correlation between SPFs and TLI on the same specimens (McDivitt et al., 1986).

The SPF values obtained in the present study are probably the lowest reported, but correspond quite reasonably to the aforementioned TLIs. It should be pointed out that 'state of

**Figure 5** DNA histogram and BrdU/DNA cytogram (both represent the total data set) of a cytological sample from an axillary lymph node. On the corresponding diagnostic slide, it was estimated to contain less than 15% tumour cells, which correlates with the hypertetraploid peak on the DNA histogram. The aneuploid BLI was estimated by setting a gate based on the DNA histogram, excluding as far as possible data points corresponding to lymphocytes.

**Figure 6** Frequency distribution of SPFs computed form DNA histograms of 142 breast cancers.

**Figure 7** Frequency distrubution of BLIs derived from BrdU/DNA cytograms of 118 breast cancers.

**Figure 8** Comparison of SPFs and BLIs obtained simultaneously on the same cytological samples for 94 patients. The straight line represents results from linear regression analysis. The vertical and horizontal lines correspond to the median SPF and BLI values. Thus only six cases were found in the lower right and upper left (discordant) quadrants.

**Figure 9** Comparison of BLIs obtained by scoring immunoperoxidase-stained nuclei on cytocentrifuged slides to those obtained by FCM. The linear regression line is superimposed to the data points. Seven of the eight points for which discordant SPF and FCM-BLI values had been obtained, appear as full squares.

**Figure 10** DNA histograms of three repetitive FNS obtained at weekly intervals from the same patient.

the art' notions in DNA-FCM, such as doublet discrimination and the use of red fluorescence peak area histograms rather than peak height (Dressler & Bartow, 1989), were applied in the present study. However, the most important correction factor was background subtraction. In this respect, our results agree with those of Haag *et al.* (1987), who were the first to apply this type of correction to DNA histograms of breast cancer specimens. In spite of these technical improvements, SPFs could be calculated for only 141 out of 176 patients (80%), and for 148 out of 183 if non-malignant specimens were included. Of the 35 cases for which no SPF could be obtained (except the case for which the quality of the histogram was judged insufficient), two corresponded to histograms with excessive debris, one to a single hypodiploid G1 peak, 16 to samples with minor aneuploid clones and 15 to multiploid tumours.

A frequent criticism with regard to SPF values has also been its inability to distinguish cells that are actively synthesising DNA from the putative S-quiescent cells (Darzynkiewicz *et al.*, 1980). BrdU incorporation has been found to correlate quite well with $^3$H-thymidine labelling index in non-Hodgkin's lymphoma (Silvestrini *et al.*, 1988). Being a non-isotopic method, BrdU labelling presents the advantage of simpler processing of specimens. We have studied BrdU incorporation in the same series of cytological samples where DNA-FCM histograms were obtained, in order to (i) increase the proportion of patients for whom proliferative status could be obtained, and (ii) compare the quantitative results of the different methods. Such samples are already dissociated, so that incubation with BrdU starts only seconds after the cells have been withdrawn from the tumour.

The ultimate goal being to introduce proliferative activity as one of the parameters used for treatment decision, a number of conditions should be met:

(1) The assay should be easily performed in routine. This was achieved here by using a short incubation time, compatible with the duration of the FNS procedure for each patient. The quality of the staining was found excellent both after immunocytochemical staining on cytocentrifuged slides and bipametric BrdU/DNA FCM-analysis. At the concentration of 30 μM BrdU, neither an increase in the volume of incubation nor a more prolonged exposure seemed to be necessary. In fact, staining was apparent within 10 min of incubation, even at room temperature. Although most cytocentrifuged samples were of good quality, the morphological distinction between normal and malignant cells was often poor on stained slides, leading possibly to an underestimation of the BLI. In the analysis of the FCM-derived BLIs, the dual-parameter cytograms allowed at least the distinction of diploid *vs* aneuploid or multiploid cell populations and their respective labelled fractions. Indeed we observed that in tumours showing both diploid and aneuploid cell populations (verified cytologically), total BLI was often reduced compared to the one computed after gating on the sole aneuploid cells. One hundred and twenty-five of the 189 cytological samples contained a sufficient number of cells for FCM-BLI analysis, 122 yielded interpretable cytograms (118 corresponding to cytologically malignant lesions). The range of

**Table II** Comparison of FCM findings in repetitive FNS

| | *Case 1* | *Case 2* | *Case 3* | *Case 4* | *Case 5* | *Case 6* | *Case 7* |
|---|---|---|---|---|---|---|---|
| | 0.98 | 1.17/1.57/2.04/2.25 | 1.71/1.86 | 1.78 | 1.65 | 1.08 | 1.00 |
| DNA index | 0.98 | (1.10)/2.20 | 1.70 | 1.80 | 1.65/1.80 | 1.08/1.88 | 1.00 |
| | 1.00 | 1.10/1.65/2.18 | 1.65/1.82 | 1.80 | 1.80 | 1.06 | 1.00 |
| | 2.7 | ND | ND | 9.2 | 4.1 | 9.8 | 1.3 |
| SPF | 2.4 | 1.8 | 3.1 | 7.8 | 2.8 | ND | 1.0 |
| | 2.5 | ND | ND | 8.5 | ND | 7.8 | 1.7 |
| | 1.9 | 2.0 | 1.5 | ND | 3.0 | ND | ND |
| BLI | 0.9 | ND | 1.4 | 1.7 | 3.1 | 3.3 | ND |
| | ND | ND | ND | 1.6 | 2.1 | 3.5 | 1.5 |

ND: not determined.

**Table III** Proliferation vs histopathological grade, SHR and DNA ploidy

| | | n | SPF % | Significance | n | BLI % | Significance |
|---|---|---|---|---|---|---|---|
| SBR Grade | I | 14 | 2.0 | Variance | 11 | 1.5 | Variance |
| | II | 64 | 3.9 | $P<0.001$ | 50 | 2.3 | $P<0.0002$ |
| | III | 23 | 6.1 | | 21 | 5.1 | |
| ER | + | 88 | 3.6 | $P<0.0001$ | 75 | 2.3 | $P<0.0001$ |
| | − | 25 | 7.3 | t-test | 21 | 5.4 | t-test |
| PgR | + | 62 | 3.5 | $P<0.0001$ | 55 | 2.4 | $P<0.002$ |
| | − | 50 | 5.9 | t-test | 42 | 4.1 | t-test |
| DNA ploidy | Diploid | 46 | 2.6 | $P<0.0001$ | 30 | 1.6 | $P<0.001$ |
| | Aneuploid | 95 | 5.4 | t-test | 88 | 3.5 | t-test |

the latter was 0.3–17.4%, with a mean of 3.0 and a median of 2.2%, values that agree quite well with TLIs in the literature, despite the very short incubation time used in our study.

(2) The information should be obtained in a large majority of patients. We have shown that for the 189 consecutive patients, both SPF and BLI could be obtained in 52%, SPF alone in 26% and BLI alone in 13%, with at least one quantitative index of proliferative activity available for 91% of the patients. However, it was still to be shown whether one of the indices was sufficient to characterise the proliferative activity of a given tumour. Indeed, the frequency distributions for SPFs and BLIs were found slightly different but the difference was limited to the 70–80th percentile. This result was relatively surprising since the values for the 94 cases for which both SPF and BLI were obtained simultaneously from dual parameter cytograms, showed a good correlation ($r = 0.85$, $P<0.0001$). A possible bias introduced by the selection of samples for FCM BLI analysis based on the number of cells in hte FNS cannot be excluded. Another explanation could reside in the fact that BLIs can be determined for cases where SPF is inapplicable. Thus each index characterises a slightly different population of breast cancers.

(3) The procedure should provide reliable results. Our study revealed that whenever SPFs and BLIs were available for the same cases, their values were closely correlated. Less than 10% discordant values were observed. However, for clinical purposes a discrete classification should be more useful than continuous values. If the patients were simply classified as above or below median, six presented discordant classification and only two of these had clearly divergent values. Thus despite the slight differences in the frequency distributions, any of the two indices of proliferative activity should provide reliable information, using the appropriate cut-off values for each distribution.

Still, two questions remained concerning the adequacy of FNS for DNA and BrdU incorporation studies. The first concerns cell death induced by FNS, but the infrequent observation of cases showing SPFs considerably higher than BLI indicates a minor influence of a possible trauma caused by the FNS. The second addressed tumour heterogeneity and the representativity of sampling relative to the tumour mass. Repeated samplings were performed on seven patients, as already attempted by others (Mullen & Miller, 1989). Modifications of the DNA histograms were observed in four out of seven, consisting in variable representation of multiple DNA peaks. Thus, multiploidy may have been underestimated in breast cancer. Although the number of patients is small, both SPF and BLI were fairly reproducible, as were the discordances between the two. This consistency of the proliferation indices, with variable representation of genetically different clones deserves further investigation regarding tissular kinetic homeostasy.

It has recently been shown that *in vivo* kinetic information can be obtained after BrdU infusion (Hoshino *et al.*, 1985; Wilson *et al.*, 1988). The latter paper has shown that by delaying the biopsy relative to the injection, not only labelling indices but also potential doubling times can be estimated from a single sample. But our method, based on FNS, at least for breast cancer patients, may be preferable because of its simplicity and its applicability without prior diagnosis of cancer. This information on tumour proliferation, available at diagnosis should prove valuable for prognosis and treatment decision.

### References

BAISCH, H., BECK, H.P., CHRISTENSEN, I.J. & 10 others (1982). A comparison of mathematical methods for the analysis of DNA histograms obtained by flow cytometry. *Cell Tissue Kinet*, **15**, 235.

BARLOGIE, B., JOHNSTON, D.A., SMALLWOOD, L. & 5 others (1982). Prognostic implications of ploidy and proliferative activity in human solid tumors. *Cancer Genet. Cytogenet.*, **6**, 17.

BLOOM, H.J. & RICHARDSON, W.W. (1957). Histological grading and prognosis in breast cancer. *Br. J. Cancer*, **11**, 359.

BRIFFOD, M., SPYRATOS, F., TUBIANA-HULIN, M. & 4 others (1989). Sequential cytopunctures during preoperative chemotherapy for primary breast cancer: cytomorphologic changes, initial tumor ploidy and tumor regression. *Cancer*, **63**, 631.

DARZYNKIEWICZ, Z., TRAGANOS, F. & MELAMED, M.R. (1980). New cell cycle compartments identified by multiparameter flow cytometry. *Cytometry*, **2**, 98.

DOLBEARE, F., GRATZNER, H., PALLAVICINI, M. & GRAY, J.W. (1983). Flow cytometric measurement of total DNA content and incorporated bromodeoxyuridine. *Proc. Natl Acad. Sci. USA*, **80**, 5573.

DRESSLER, L.G., SEANER, L.C., OWENS, M.A., CLARK, G.M. & MCGUIRE, W.L. (1988). DNA flow cytometry and prognostic factors in 1331 frozen breast cancer specimens. *Cancer*, **61**, 420.

DRESSLER, L.G. & BARTOW, S.A. (1989). DNA flow cytometry in solid tumors: practical aspects and clinical applications. *Sem. Diag. Pathol.*, **6**, 55.

HAAG, D., FEICHTER, G., GOERTTLER, K. & KAUFMAN, M. (1987). Influence of systematic errors on the evaluation of the S-phase portions from DNA distributions of solid tumors as shown for 328 breast carcinomas. *Cytometry*, **8**, 377.

HEDLEY, D.W., RUGG, C.A. & GELBER, R.D. (1987). Association of DNA-index and S-phase fraction with prognosis of node positive early breast cancer. *Cancer Res.*, **47**, 4729.

HERY, M., GIOANNI, J., LALANNE, C.M., NAMER, M. & COURDI, A. (1987). The DNA labelling index: a prognostic factor in node negative breast cancer. *Br. Cancer Res. Treat.*, **6**, 207.

HOSHINO, T., NAGASHIMA, T., MUROVIC, J., LEVIN, E.M., LEVIN, V.A. & RUPP, S.M. (1985). Cell kinetic studies of in situ human brain tumors with bromodeoxyuridine. *Cytometry*, **6**, 627.

KALLIONEMI, O.P., HIETANEN, T., MATTILA, J., LEHTINEN, M., LAUSLAHTI, K. & KOIVULA, T. (1987). Aneuploid DNA content and high S-phase fraction of tumour cells are related to poor prognosis in patients with primary breast cancer. *Eur. J. Cancer Clin. Oncol.*, **23**, 277.

KALLIONIEMI, O.P., BLANCO, G., ALAVAIKKO, M. & 5 others (1988). Improving the prognostic value of DNA flow cytometry in breast cancer by combining DNA index and S-phase fraction. *Cancer*, **62**, 2183.

KUTE, T.E., MUSS, H.B., HOPKINS, M., MARSHALL, R., CASE, D. & KAMMIRE, L. (1985). Relationships of flow cytometry results to clinical abd steroid receptor status in human breast cancer. *Breast Cancer Res. Treat.*, **6**, 113.

MAGDELENAT, H., LAINE-BIDRON, C., MERLE, S. & ZAJDELA, A. (1987*a*). Estrogen and Progestin receptor assay in fine needle aspirates of breast cancer: methodological aspects. *Eur. J. Cancer Clin. Oncol.*, **23**, 425.

MAGDELENAT, H., MERLE, S. & ZAJDELA, A. (1987*b*). Enzyme ummunoassay of estrogen receptors in fine needle aspirates of breast tumors. *Cancer Res.*, **47**, 4265s.

MCDIVITT, R.W., STONE, K.R., CRAIG, B.R., PALMER, J.O., MEYER, J.S. & BAUER, W.C. (1986). A proposed classification of breast cancer based on kinetic information. *Cancer*, **57**, 269.

MEYER, J.S. (1986). Cell kinetics in selection and stratification of patients for adjuvant therapy of breast carcinoma. *NCI Monograph*, **1**, 25.

MEYER, J.S., MCDIVITT, R.W., STONE, K.R., PREY, M.U. & BAUER, W.C. (1984). Practical, breast carcinoma cell kinetics: review and update. *Breast Cancer Res. Treat.*, **4**, 79.

MULLEN, P. & MILLER, W.R. (1989). Variations associated with the DNA analysis of multiple fine needle aspirates obtained from breast cancer patients. *Br. J. Cancer*, **59**, 688.

NORDENSKJÖLD, B., LÖWHAGEN, T., WERSTERBERG, H. & ZAJICEK, J. (1976). $^{3}$H Thymidine incorporation into mammary carcinoma cells obtained by needle aspiration before and during endocrine therapy. *Acta Cytol.*, **20**, 137.

REMVIKOS, Y., MAGDELÉNAT, H. & ZAJDELA, A. (1988). DNA flow cytometry applied to fine needle samplings of human breast cancer. *Cancer*, **61**, 1629.

REMVIKOS, Y., BEUZEBOC, P., ZAJDELA, A., VOILLEMOT, N., MAGDELÉNAT, H. & POUILLART, P. (1989). Pretreatment proliferative activity of breast cancer correlates with the response to cytotoxic chemotherapy. *J. Natl Cancer Inst.*, **18**, 1383.

SCHUTTE, B., REYNDERS, M.M.J., VAN ASSCHE, C.L.M.V., HUPPERETS, P.S.G.J., BOSMAN, F.T. & BLIJHUM, G.H. (1987). An improved method for the immunocytochemical detection of bromodeoxyuridine labeled nuclei using flow cytometry. *Cytometry*, **8**, 372.

SILVESTRINI, R., DAIDONE, M.G. & GASPARINI, G. (1985). Cell kinetics as a prognostic marker in node-negative breast cancer. *Cancer*, **56**, 1982.

SILVESTRINI, R., COSTA, A., VENERONI, S., DEL BINO, G. & PERSICI, P. (1988). Comparative analysis of different approaches to investigate cell kinetics. *Cell Tissue Kinet.*, **21**, 123.

SPYRATOS, F., BRIFFOD, M., GENTILE, A., BRUNET, M., BRAULT, C. & DESPLACES, A. (1987). Flow cytometric study of DNA distribution in cytopunctures of benign and malignant breast lesions. *Analyt. Quant. Cytol. Histol.*, **9**, 486.

TUBIANA, M., PEJOVIC, M.H., CHAVAUDRA, N., CONTESSO, G. & MALAISE, E.P. (1984). The long-term prognostic significance of the thymidine labelling index in breast cancer. *Int. J. Cancer*, **33**, 441.

WILSON, G.D., MCNALLY, N.J., DISHE, S. & 4 others (1988). Measurement of cell kinetics in human tumours *in vitro* using bromodeoxyuridine incorporation and flow cytometry. *Br. J. Cancer*, **58**, 423.

ZAJDELA, A., ZILHARDT, P. & VOILLEMOT, N. (1987). Cytological diagnosis by fine needle sampling without aspiration. *Cancer*, **59**, 1201.